# Bioinformatic analysis and clinical diagnostic value of hsa_circ_0004099 in acute ischemic stroke

**DOI:** 10.1371/journal.pone.0277832

**Published:** 2022-11-18

**Authors:** Jiqing Zheng, Shuiming Luo, Yaobin Long

**Affiliations:** Deparment of Rehabilitation, The Second Affiliated Hospital of Guangxi Medical University, Nanning, Guangxi, China; Indiana University Purdue University at Indianapolis, UNITED STATES

## Abstract

This study investigates the expression and effect of hsa_circ_0004099 in acute ischemic stroke (AIS). We conducted a case-controlled study that included 40 patients with AIS within 24 hours and 40 healthy subjects during the same period as a control group. Differentially expressed circular RNAs (circRNAs) were obtained using GEO2R, and the expression of hsa_circ_0004099 was verified using RT-PCR. Correlation analysis of the National Institutes of Health Stroke Scale (NIHSS) disease severity score and ischemic time with hsa_circ_0004099 expression levels was also performed. The receiver operating characteristic (ROC) curve of hsa_circ_0004099 was constructed, and bioinformatic analysis of hsa_circ_0004099 was performed. NIHSS scores negatively correlated with hsa_circ_0004099 levels (P<0.001, r = -0.7053), whereas infarct time was negatively correlated with hsa_circ_0004099 levels (P<0.001, r = -0.5130); hsa_circ_0004099 could benefit clinical diagnosis (area under the curve [AUC]: 0.923 [95% confidence interval [CI]: 0.8680–0.9904]). Kyoto encyclopedia of genes and genomes (KEGG) analysis showed that hsa_circ_0004099 was enriched in several cancer pathways, which were collectively enriched in four genes namely TCF7L2, NRAS, CTNNB1, and KRAS. Eight core proteins were screened using a protein-protein interaction (PPI) network namely SMAD4, HIF1A, CTNNB1, CDKN1B, CDK6, FOXO3, KRAS, and NRAS. hsa_circ_0004099 is a potential clinical diagnostic marker. In addition, the possible role of hsa_circ_0004099 in the pathogenesis of AIS was analyzed using bioinformatics, which provided a new potential molecular target for AIS treatment.

## Introduction

Eighty-five percent of stroke survivors experience an acute ischemic stroke (AIS) caused by intracranial artery occlusion or extracranial carotid artery occlusion, leading to brain tissue death, focal neurological deficits, and disability, thereby causing a huge burden to patients and society [1, 2]. Mechanical thrombectomy and tissue plasminogen activator are the most popular treatment strategies against AIS. However, there are limitations in the time window for treatment and problems with nerve cell ischemia-reperfusion injury [3]. The most commonly used diagnostic tools for AIS are CT and MRI. However, MRI is not suitable for patients with metal stents and is expensive [4]. Additionally, in the early stages of the disease, CT cannot identify symptoms in 40–50% of patients with AIS [5]. Therefore, there is a need to identify sensitive, reliable, and affordable biomarkers for early screening and diagnosis as well as effective therapeutic targets.

Circular RNAs (circRNAs) are a class of noncoding RNAs widely present in eukaryotic cells [6]. Unlike linear RNA with a 5 ’end cap and 3’ poly-a tail, circRNA has a covalently closed loop structure [7]. The covalent bond within the circRNA loop is resistant to ribonuclease degradation, ensuring structural stability and expression conservation [8]. In recent years, with the development of circRNA detection technology, some circRNAs have shown potential properties as biomarkers for AIS using high-throughput sequencing [9–13].

The host gene of hsa_circ_0004099 is differentially expressed in normal and neoplastic cells (DENN) domain-containing 5A (DENND5A) gene. The DENN domain is an evolutionarily ancient enzymatic module that confers guanine nucleotide exchange factor activity to proteins and is a key regulator of cell membrane trafficking, mainly in neuronal tissues. Knockdown of DENND5A results in marked alterations in neuronal development [14]. In this study, we used GEO2R to obtain differentially expressed circRNAs in the AIS plasma circRNA chip, and found that the expression of hsa_circ_0004099 was downregulated, while its log2FC absolute value (3.52) was the largest among the downregulated circRNAs. To date, no clinical studies have evaluated the role of hsa_circ_0004099 in AIS. Therefore, in this study, correlation between hsa_circ_0004099 expression and AIS clinical indicators was first explored, followed by bioinformatic analysis of hsa_circ_0004099 in AIS, to provide new potential molecular targets for AIS treatment.

## Materials and methods

### General clinical data

This was a case-controlled study. Forty AIS patients were recruited from the emergency department of the Second Affiliated Hospital of Guangxi Medical University from April 2021 to August 2021. In addition, 40 healthy subjects matched for gender and age with patients with AIS were recruited in the same hospital and physical examination center during the same period. This study was approved by the Ethics Committee of the Second Affiliated Hospital of Guangxi Medical University (NO:2021 [KY-011]) and all subjects provided written informed consent. Patients with MRI- and CT-confirmed AIS were included in the study. The inclusion and exclusion criteria were as follows: Inclusion criteria: 1) Aged 55–75 years old; 2) AIS occurred for the first time; 3) Patients admitted within 24 hours. Exclusion criteria: 1) AIS complicated with other cerebrovascular diseases; 2) patients with severe infection or taking anticoagulant drugs at the time of admission; 3) patients who received any AIS intervention therapy. Whole blood was collected from all subjects in two 5 ml EDTA anticoagulation tubes.

### Validation of the expression of hsa_circ_0004099 in human plasma

Five milliliters of whole blood from an EDTA anticoagulation tube were collected within 24 hours after admission to the emergency department. The blood was then kept for 1 h and centrifuged at 4,000 × *g* for plasma extraction. Each sample consumed 250 μl plasma for total RNA extraction. The concentration of total RNA in each sample was greater than 100 ng/μl, and the ratio of A260/280 was between 1.8–2.0. cDNA was reverse transcribed into cDNA using the HiScript Ill RT SuperMix for gPCR (genomic DNA [gDNA] wiper) reverse transcription kit (Vazyme Biotech Co., Ltd). The reaction conditions were as follows: 42°C for 2 min → 4°C hold (removal of gDNA); 37°C for 15 min → 85°C for 5 s → 4°C (reverse transcription). A ChamQ Universal SYBR qPCR Master Mix [Vazyme Biotech Co., Ltd.] was used to quantify the expression levels of hsa_circ_0004099. The reaction conditions were as follows: pre-denaturation at 95°C for 30 s → 40 cycles of 95°C for 10 s, and 60°C for 30 s. Primer design and synthesis were performed using the Takara Bio software (Table 1). Data were analyzed using the 2^-△△Ct^ method.

**Table 1:** Primer sequences.

Gene	Primer	Sequence
GADPH	forward	5′-GCACCGTCAAGGCTGAGAAC-3′
	reverse	5′-TGGTGAAGACGCCAGTGGA-3′
hsa_circ_0004099	forward	5′-GACATTTGGGTTTGCCCTCAC-3′
	reverse	5′-TCCAGCACGCTCCGACAT-3′

### Data collection

For the veracity of the data collected, clinical data of AIS patients and healthy subjects were collected by non-authors of this study from April 2021 to February 2022, and the study authors were only allowed access to participant-specific information after data collection was complete. This information includes gender, age, smoking, hypertension, and diabetes in both AIS patients and healthy subjects. In addition, the National Institutes of Health Stroke Scale (NIHSS) disease severity score was recorded within 24 hour of admission for AIS. Furthermore, patients were classified according to the cerebral infarction etiological classification (Trial of Org 10172 in Acute Stroke Treatment, TOAST) criteria.

### Bioinformatic analysis

The human plasma circRNA gene assay was selected from the GEO database (https://www. ncbi. nlm. nih. gov/), and platform files as well as a series of matrix files were downloaded. circRNA definition conditions were as follows: |log2FC|≥2, p <0.05.

The microRNAs (miRNAs) bound by circRNAs were predicted using the circular RNA Interactom (https://circinteractome.nia.nih.gov/) and circBank (http://www.circbank.cn/). Next, the intersection of miRNAs was obtained. TargetScan (http://www.targetscan.org/vert_72/), miRDB (http://mirdb.org/index.html), and miRTarbase (https://mirtarbase.cuhk.edu.cn/) predicted the downstream target genes of intersecting miRNAs, and the intersecting results of these three software programs were considered co-regulated mRNAs. Finally, a Venn diagram of the intersection of miRNAs and mRNAs was constructed using a free online platform for data analysis and visualization (http://www.bioinformatics.com.cn).

Visual analysis of circRNA-miRNA-mRNA networks was performed using Cytoscape 3.7.2. Gene ontology (GO) and Kyoto encyclopedia of genes and genomes (KEGG) enrichment analysis of hsa_circ_0004099 downstream target genes were performed using DAVID (https://david.ncifcrf.gov/).

Furthermore, a bubble map and KEGG signaling pathway map were obtained using an online platform for data analysis and visualization (http://www.bioinformatics.com.cn). The target genes were obtained in TSV format using STRING (https://string-db.org/) and imported into Cytoscape. Finally, a protein-protein interaction (PPI) network map was obtained using the NetworkAnalyzer degree algorithm and the combined score.

### Statistical analysis

Statistical data were collected using GraphPad Prism 8.0.1 and SPSS 23. The measurement data are expressed as $\bar{x}$ ± s or median and quartile range (IQR). According to the data distribution, the two groups were compared using the single-sample t-test or Mann-Whitney *U*-test, multi-group comparisons were performed using single-factor analysis or the Kruskal-Wallis H-test, and pairwise comparisons were performed using the Student–Newman–Keuls (S-N-K) test or Steel-Dwass test. In the correlation analysis, Pearson linear correlation was used for measurement data, Spearman rank correlation was used for grade data, and a receiver operating characteristic (ROC) curve was drawn to demonstrate the diagnostic ability of hsa_circ_0004099. A P-value of < 0.05 was considered statistically significant.

**Fig 1:**
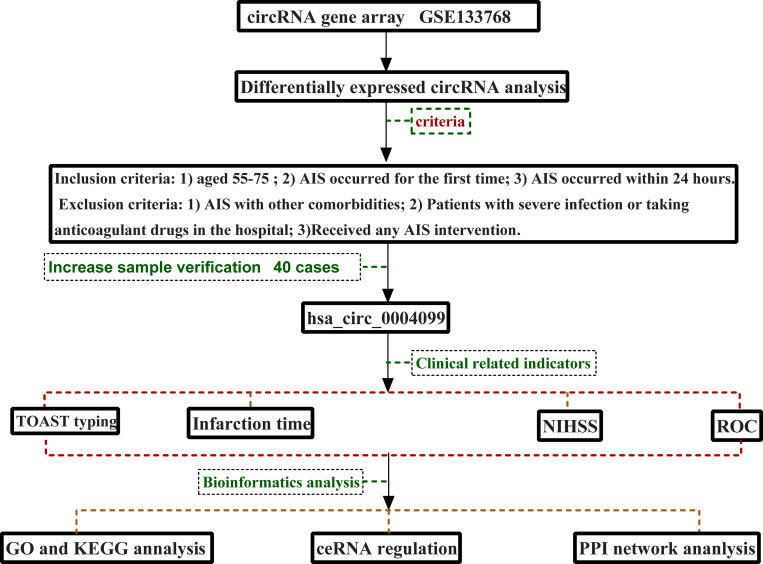
Experimental technical route.

This study strictly followed the above process (Fig 1).

## Results

### Clinical baseline characteristics of patients with AIS and healthy subjects

All participants completed the study. Table 2 lists the demographic information and clinical characteristics of all subjects. There was no statistical difference between patients with AIS and healthy subjects in terms of age (P = 0.239), sex (P = 0.813), smoking (P = 0.651), hypertension (P = 0.116), and diabetes (P-0.204). NIHSS scores were divided into three grades (mild: NIHSS < 4; moderate: 5 ≤ NIHSS < 15; severe: NIHSS ≥ 15) [15]. Among all patients with AIS in this study, there were 12 mild, 22 moderate, and 6 severe cases. In addition, stroke subtypes were divided into five categories according to TOAST criteria: (1) large-artery arteriosclerosis (LAA), (2) small-artery occlusion (SAO), (3) cardiac-origin cardioembolism (CE), (4) stroke of other confirmed etiology, and (5) stroke of undetermined etiology [16]. The patients in this study had three stroke subtypes, namely, LAA (n = 17), SAO (n = 15), and CE (n = 8).

**Table 2:** Clinical data characteristics of the experimental group and control group.

Characteristics	AIS group			Healthy group	P
	n = 40			n = 40	
Age (years) (x±*s*)	63.60±6.18			60.90±5.45	0.239
Gender, n (%)					0.813
Male	26 (65%)			27 (67.5%)	
Female	14 (35%)			13 (32.5%)	
Smoke, n (%)	18 (45%)			16 (40%)	0.651
Hypertension, n (%)	25 (62.5%)			18 (45%)	0.116
Diabetes mellitus, n (%)	13 (32.5%)			8 (20%)	0.204
NIHSS Degree Grading (Cases)	Mild	Moderate	Severe	--	--
	12	22	6		
TOAST (Cases)	SAO	LAA	CE	--	--
	15	17	8		

### Expression of hsa_circ_0004099 and analysis results of clinically relevant indicators

**Fig 2:**
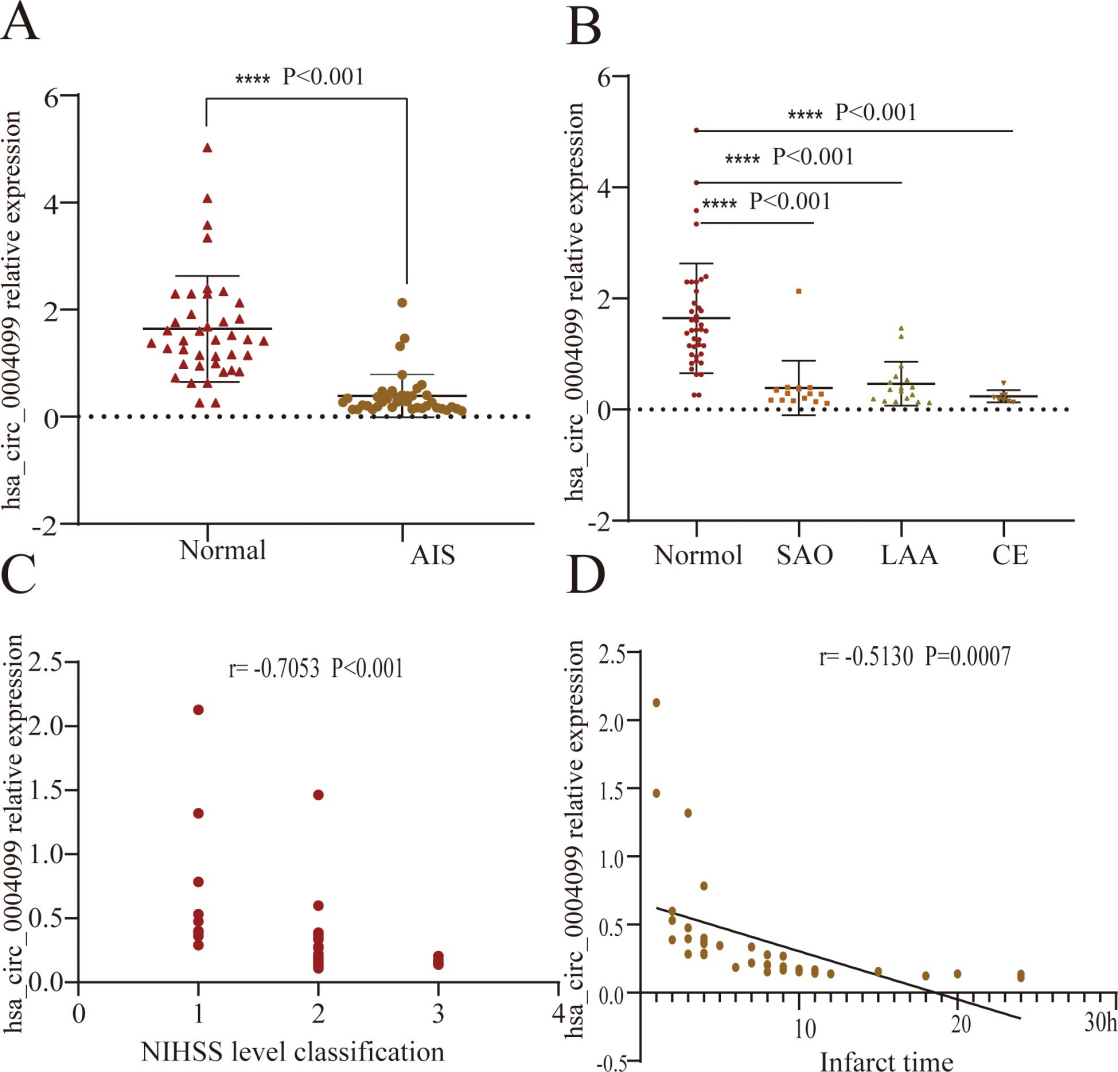
Expression levels of hsa_circ_0004099 in healthy subjects and patients with AIS. **A:** The Mann-Whitney *U*-test was used for two-sample comparisons. The expression level of hsa_circ_0004099 was lower in patients with AIS compared with that in healthy subjects. **B:** The Steel-Dwass test was used for pairwise comparisons. The levels of hsa_circ_0004099 in patients with small-artery occlusion (SAO), large-artery arteriosclerosis (LAA), and cardiac-origin cardioembolism (CE) were different compared to those of normal individuals (P<0.001). **C:** Comparison of National Institutes of Health Stroke Scale (NIHSS) disease severity scores and hsa_circ_0004099 levels using Spearman rank correlation: 1 on the abscissa represents "mild,” 2 represents "moderate,” and 3 represents "severe." The expression level of hsa_circ_0004099 showed a downward trend and was highly negatively correlated with the NIHSS scores (P<0.001, r = -0.7053). **D:** Pearson correlation analysis was used to compare the infarct time with the hsa_circ_0004099 levels: 1 grid on the abscissa represents 1 hour, and each circle represents a patient with AIS (40 patients in total). As shown in the figure, with the prolongation of infarction time, the expression of hsa_circ_0004099 showed a downward trend. The levels of hsa_circ_0004099 were negatively correlated with the infarct time (P<0.001, r = -5130).

The expression level of hsa_circ_0004099 in patients with AIS (0.28 [0.17–0.40]) was lower than that in normal individuals (1.43 [0.99–2.08]), as determined using RT-PCR verification (Fig 2A), consistent with the expression level of hsa_circ_0004099 in the circRNA gene array.

Spearman rank correlation analysis showed that the severity of AIS was highly negatively correlated with the expression level of hsa_circ_0004099 (P<0.001, r = -0.7053) (Fig 2B). The expression level of hsa_circ_0004099 decreased with increasing AIS injury. This suggests that the expression level of hsa_circ_0004099 reflects AIS severity, to some extent. Pearson correlation analysis showed that the time of infarction was negatively correlated with the expression level of hsa_circ_0004099 in patients with AIS (P<0.001, r = -5130) (Fig 2C). The expression level of hsa_circ_0004099 decreased with increasing time of infarction, indicating that the expression level of hsa_circ_0004099 may be correlated with the time of cerebral infarction.

Due to the skewed distribution of the data, the Kruskal-Wallis H-test was used to compare the hsa_circ_0004099 expression levels in patients with the three infarct types, LAA, SOA, and CE, with those of healthy subjects, and pairwise analysis was performed using the Steel-Dwass test. The results showed that the hsa_circ_0004099 expression levels of patients with LAA (0.36[0.17–0.57]), SAO (0.28[0.17–0.38]), and CE (0.20[0.16–0.28]) were all significantly different from those of healthy subjects (1.43[0.99–2.08]) (P <0.001), with the expression level being lower than that of healthy subjects. There was no statistically significant difference in the expression levels of hsa_circ_0004099 among the LAA, SAO, and CE groups (Fig 2D).

#### Diagnostic value of plasma hsa_circ_0004099 levels in patie-nts with AIS

**Fig 3:**
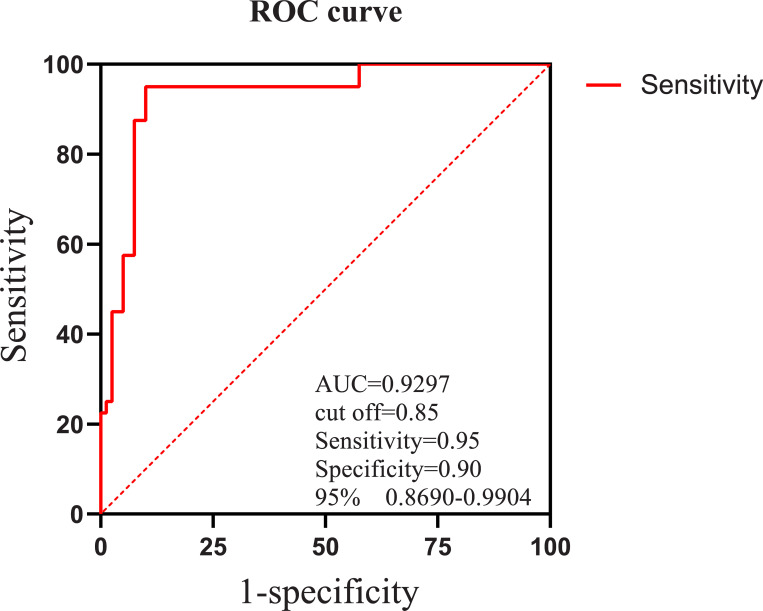
Receiver operating characteristic (ROC) curve. The abscissa indicates the sensitivity, while the ordinate indicates 1-specificity. At a cut-off of 0.85, the area under the curve (AUC) peaked, while the sensitivity and specificity of the best cut-off point were 0.95 and 0.90, respectively. At this time, the hsa_circ_0004099 level was the highest predictive value for AIS risk.

ROC curve analysis revealed that the expression levels of hsa_circ_0004099 had a good diagnostic value for AIS (area under the curve [AUC]: 0.923 [95% confidence interval [CI]: 0.8680–0.9904]). In addition, the AUC reached a maximum when the cut-off was 0.85, and the sensitivity and specificity of the optimal cut-off point were 0.95 and 0.90, respectively (Fig 3).

### hsa_circ_0004099 bioinformatic analysis results

**Fig 4:**
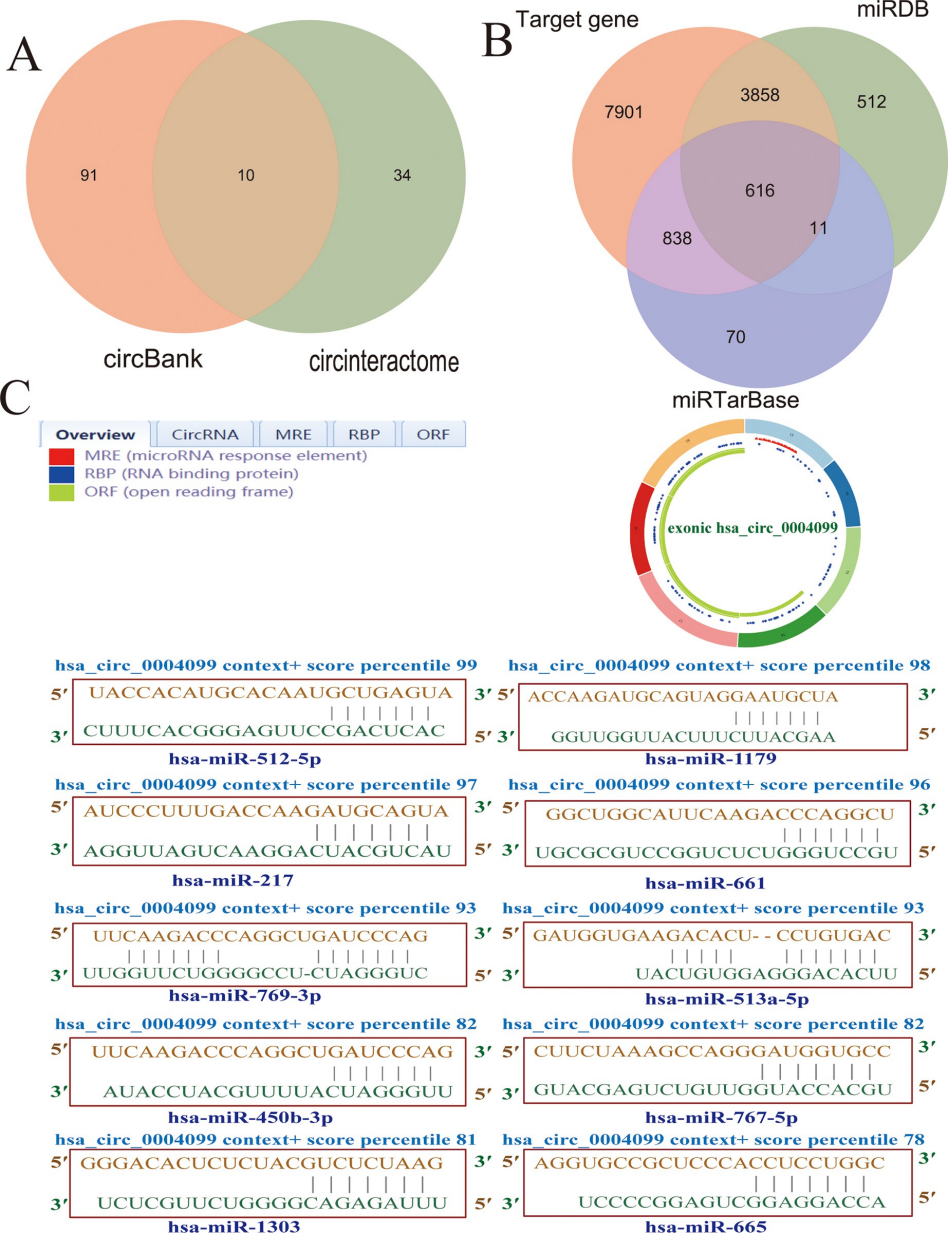
Downstream of hsa_circ_0004099 intersecting microRNAs (miRNAs) and mRNAs. **A:** Ten intersecting miRNAs predicted using the circular RNA Interactom and circBank. **B:** Ten miRNAs predicted 616 intersecting mRNAs. **C:** CSCD identified the miRNA-response elements (MRE), RNA-binding proteins (RBP), and open reading frames (ORF) of hsa_circ_0004099, which obtained the binding score of hsa_circ_0004099 and the intersection of 10 miRNAs; the closer the value was to 100, the higher was the confidence.

Ten miRNAs were found at the intersection of hsa_circ_0004099 (Fig 4A) with 616 downstream target genes (Fig 4B). In addition, the miRNA-response elements (MRE), RNA-binding proteins (RBP), and open reading frames (ORF) of hsa_circ_0004099 were obtained using CSCD (http://gb.whu.edu.cn/CSCD/). The binding fractions of hsa_circ_0004099 and of the 10 miRNAs were obtained using the circular RNA Interactom (Fig 4C).

**Fig 5:**
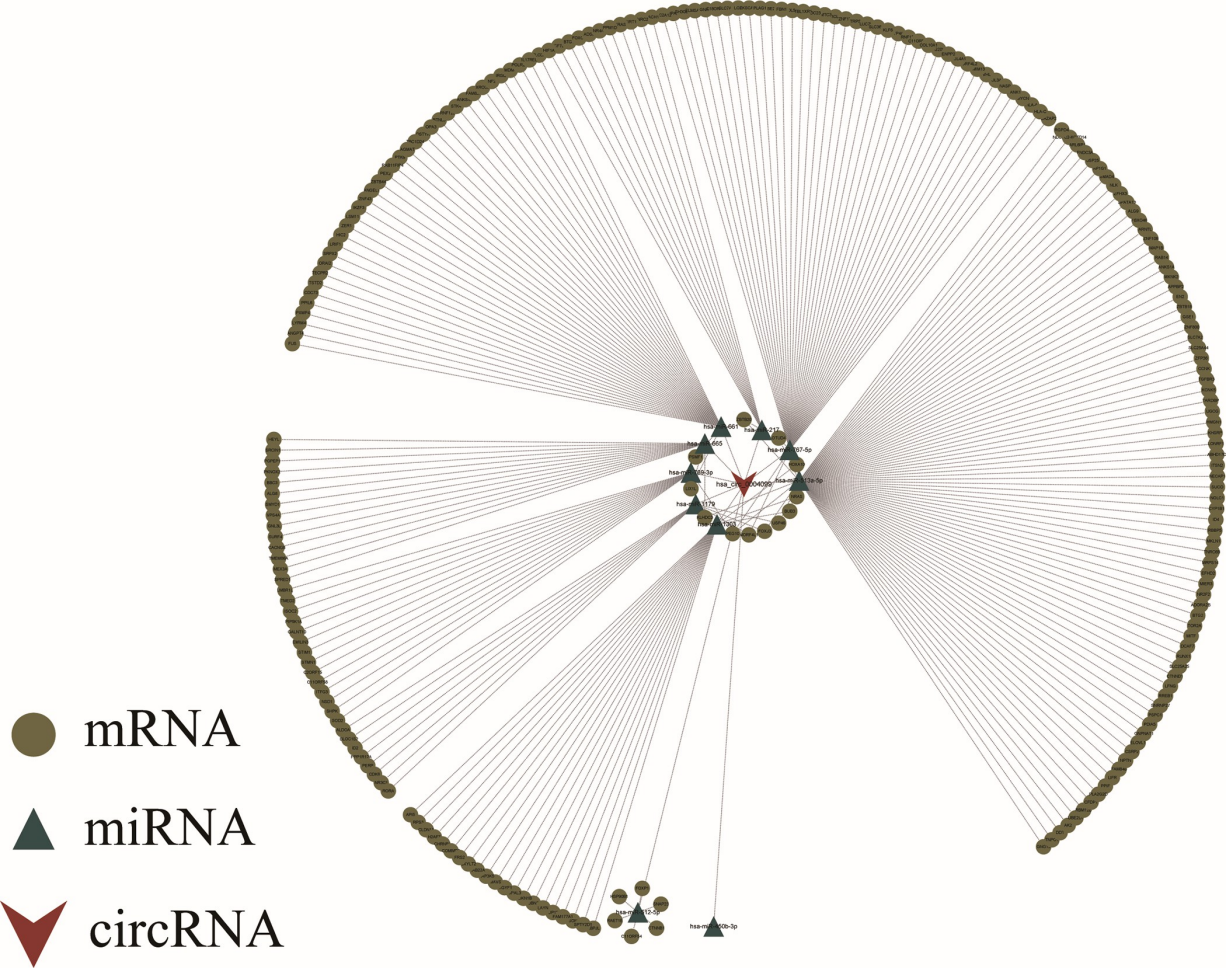
Circular RNA-microRNA-mRNA (circRNA-miRNA-mRNA) network. Inverted triangles represent hsa_circ_0004099, triangles represent miRNAs, while circles represent mRNAs.

Cytoscape was used to construct the competing endogenous RNA (ceRNA) network for hsa_circ_0004099 (Fig 5).

**Fig 6:**
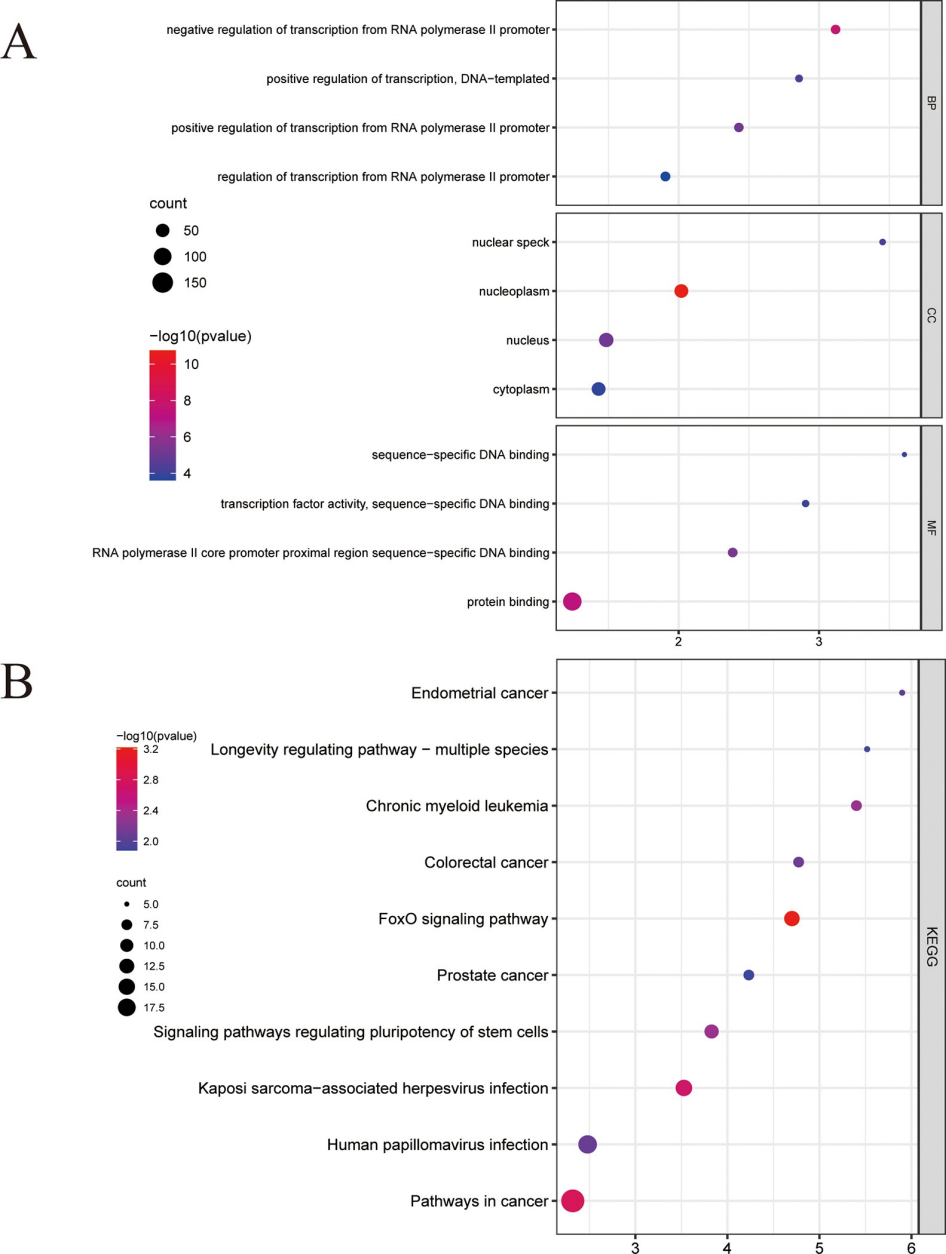
DAVID enrichment analysis. A: GO enrichment analysis: BP, biological process; CC, cellular component; MF, molecular function. The abscissa represents the number of enriched genes, while the ordinate describes the enriched items. The darker the bar color, the more significant the difference between the enriched items. **B:** KEGG enrichment analysis: the abscissa represents the number of enriched genes, while the ordinate represents the enriched pathway entries. Many cancer pathways and some common pathways are shown. The size of the circle represents the number of enriched genes: the larger the circle, the higher the number of enriched genes, and the redder the color, the closer the relationship with the enriched entry.

**Fig 7:**
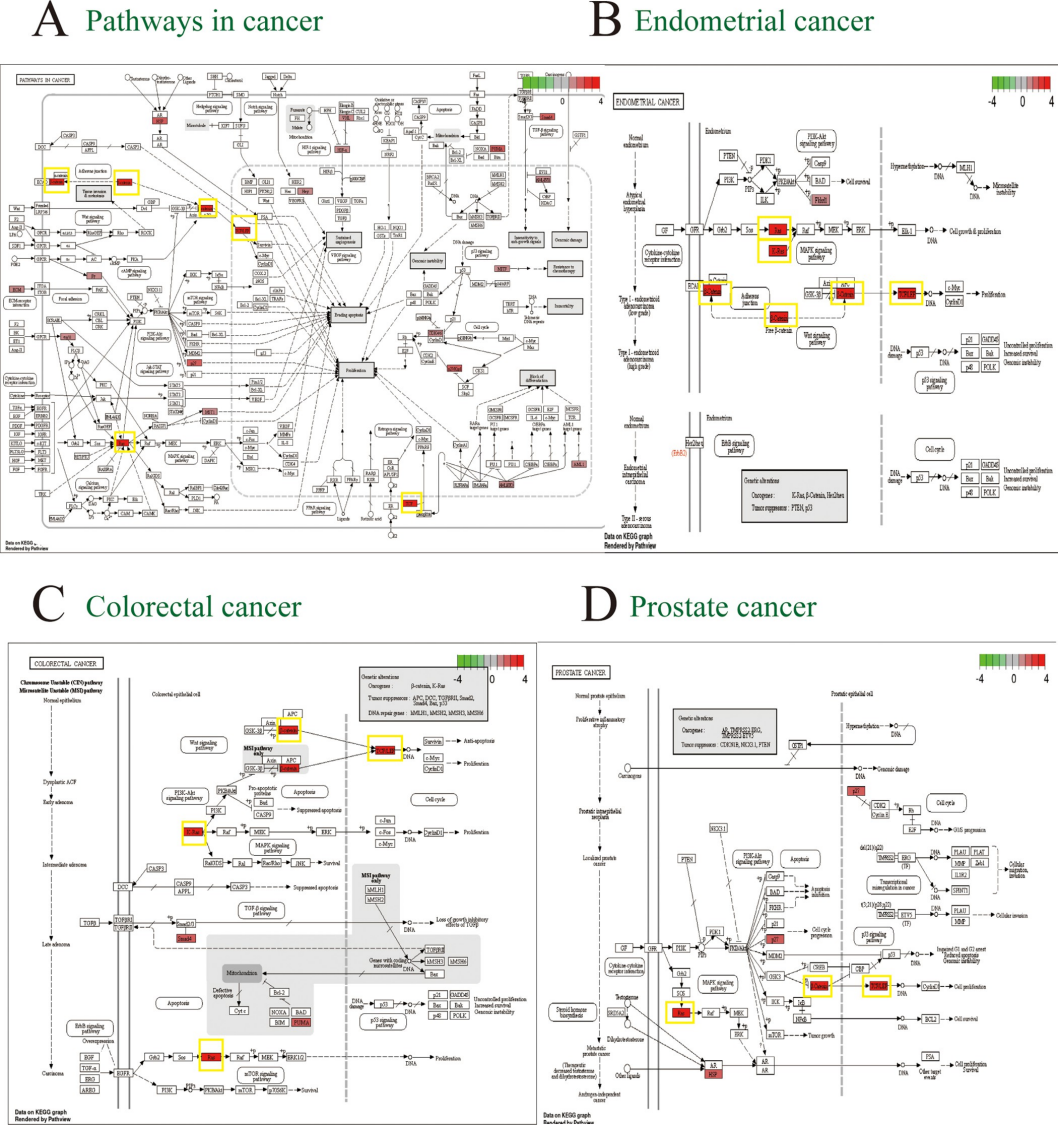
Kyoto encyclopedia of genes and genomes (KEGG) signaling pathway map. Red indicates genes that are enriched in cancer signaling pathways. The four genes, TCF7L2, NRAS, CTNNB1, and KRAS, framed in yellow are jointly enriched by these two pathways and are mainly involved in cell proliferation.

GO and KEGG enrichment analyses of target genes regulated by hsa_circ_0004099 were performed. The GO analysis results showed that the target genes were: 1) mainly enriched (Fig 6A) in the biological processes of transcriptional regulation, apoptosis, and other pathways; 2) significantly enriched in cellular components, such as nucleoplasm, cytoplasm, and nucleus; 3) significantly enriched in the molecular functions of protein binding, DNA binding, and protein kinase binding. KEGG enrichment results showed target genes (Fig 6B) mainly enriched in cancer pathways. Furthermore, these cancer pathways were jointly enriched in four genes, TCF7L2, NRAS, CTNNB1, and KRAS, which are mainly involved in cell proliferation (Fig 7).

**Fig 8:**
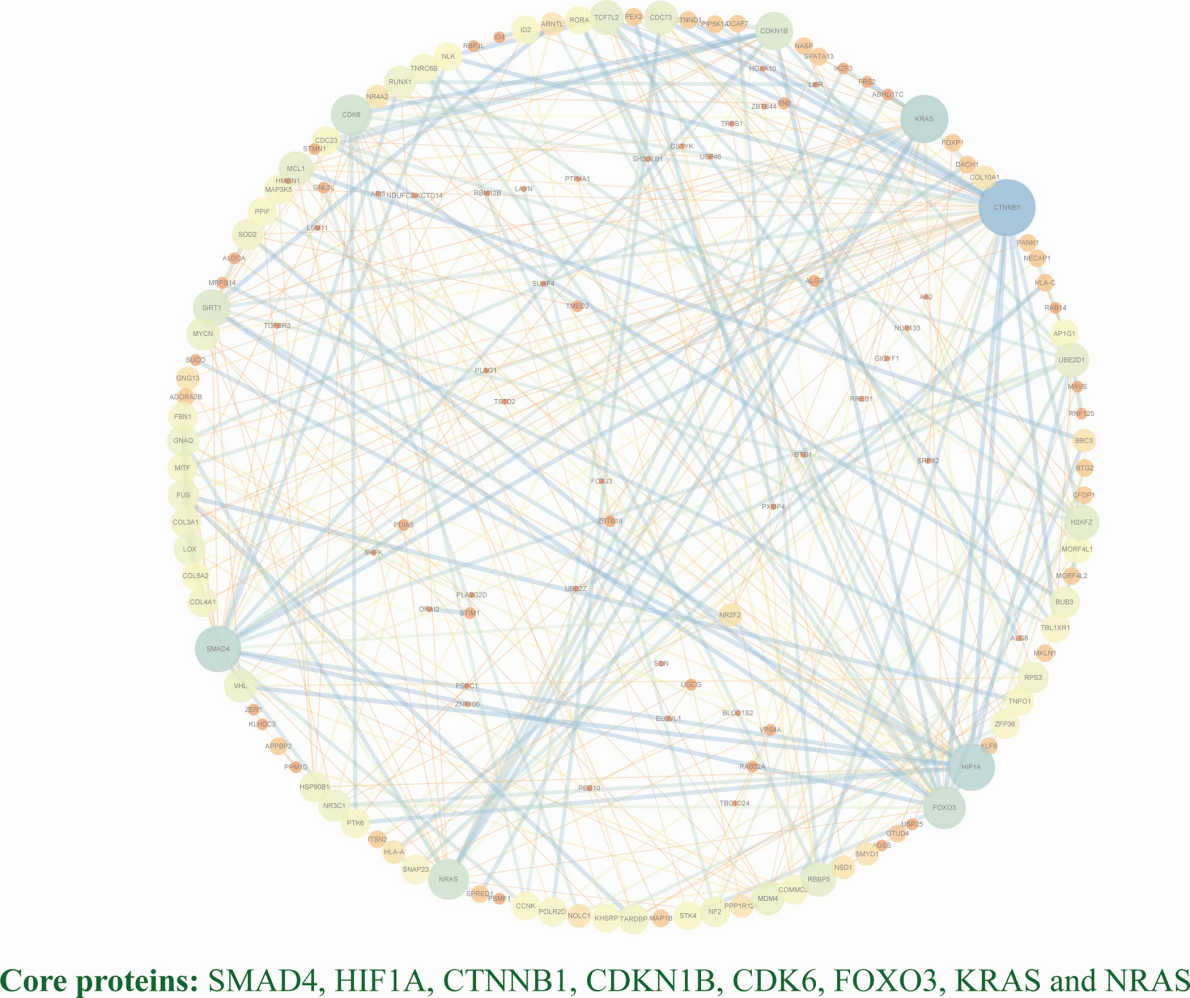
Protein-protein interaction (PPI) network. SMAD4, HIF1A, CTNNB1, CDKN1B, CDK6, FOXO3, KRAS and NRAS are the group of core proteins screened using NetworkAnalyzer.

In the PPI network, 152 nodes and 367 correlation pairs were identified. In addition, eight core proteins were present in the PPI network: SMAD4, HIF1A, CTNNB1, CDKN1B, CDK6, FOXO3, KRAS, and NRAS (Fig 8).

## Discussion

The expression of circRNAs in brain tissue is closely associated with cerebral ischemia; these circRNAs can be repeatedly detected in human peripheral blood samples [17]. The host gene of hsa_circ_0004099 is DENND5A, primarily expressed in neuronal tissues and its knockout leads to significant changes in neuronal development [14]. The expression of hsa_circ_0004099 in AIS and its clinical diagnostic properties have not been reported. In this study, RT-PCR was used to verify the expression of hsa_circ_0004099 in patients with AIS. The high-throughput sequencing results showed lower expression of hsa_circ_0004099 in patients with AIS compared with that in healthy subjects. Furthermore, the correlation between the expression of hsa_circ_0004099 and clinical indicators of AIS was explored. Moreover, the possible roles of hsa_circ_0004099 in regulating the pathogenesis of AIS were analyzed using bioinformatics, providing new potential molecular targets for AIS therapy.

The NIHSS total score ranges from 0 to 42, with higher scores associated with poor long-term prognoses [18]. In this study, NIHSS scores were highly positively correlated with hsa_circ_0004099 levels (P<0.001, r = -0.7053). The expression level of hsa_circ_0004099 increased with the decrease in AIS injury, indicating that the expression level of hsa_circ_0004099 may reflect the severity of AIS to a certain extent. Recently, Peng et al. [19] explored the expression of circRNA-HECTD1 in the peripheral blood mononuclear cells of 160 AIS patients and 160 healthy individuals using q-PCR and used the NIHSS score to evaluate the disease severity of AIS patients. The expression of circRNA-HECTD1 and NIHSS scores showed a moderate positive correlation (r = 0.462, P<0.001). Moreover, Chen et al. [20] investigated the expression of hsa_circ_014172 in the serum of patients with acute cerebral infarction. The authors found that the serum level of hsa_circ_0141720 was positively correlated with NIHSS scores (r = 0.723, p<0.001), and that NIHSS scores were positively correlated with the infarct volume of patients, indicating that the higher the score, the more severe the cerebral infarction.

In this study, the Kruskal-Wallis H rank-sum test was used to analyze the expression of hsa_circ_0004099 in patients with LAA, SOA, and CE infarction types and compare it with that in normal plasma. The expression levels of hsa_circ_0004099 in patients with LAA, SAO, and CE were significantly higher than those in normal subjects (P<0.001). In addition, most patients had LAA (17 cases), followed by SOA (15 cases) and CE (8 cases). In 2702 patients with AIS, the most common infarction subtype was LAA (39%), followed by SVO (23%) and CE (22%); LAA was the most common subtype of posterior circulation infarction [16]. These results are consistent with the etiological classification rates in this study, proving that LAA and SVO are the most common types of AIS.

The ROC curve analysis in this study indicated that the AUC of hsa_circ_0004099 to distinguish AIS patients from normal subjects was 92.3%. In addition, when the critical value of hsa_circ_0004099 was 0.85, AUC reached the maximum value, and the sensitivity and specificity of the optimal cut-off point were 0.95 and 0.90, respectively, indicating high diagnostic efficiency. In addition, Zuo et al. [21] investigated the levels of three circRNAs in the plasma of patients with AIS and healthy human plasma and found that the three circRNAs combined may have better diagnostic efficacy compared with that of each individual circRNA. This was because the AUC was 0.875, the specificity was 91%, and the sensitivity was 71.5%; however, when a single circRNA was analyzed using ROC, the diagnostic level decreased. In the future, hsa_circ_0004099 and the above-mentioned circRNAs may contribute to the diagnostic value of ROC analysis for AIS.

In the biological information section, 10 miRNAs in the downstream intersection of hsa_circ_0004099 (hsa-miR-1179, hsa-miR-1303, hsa-miR-217, hsa-miR-450b-3p, hsa-miR-769-3p, hsa-miR-512-5p, hsa-miR-513a-5p, hsa-miR-661, hsa-miR-665, and hsa-miR-767-5) were jointly predicted using the circRNA interatom and circBank. Some studys found that miR-217 and miR-665 were associated with cerebral ischemia [22–25]. miR-217 is localized on chromosome 2 and plays an important role in angiogenesis, protein acetylation, and cellular senescence (https://www.genecards.org/cgi-bin/carddis.pl?gene=MIR217#localization). Shi et al. [22] found that myocyte enhancer factor 2D (MEF2D) is a direct target of miR-217, which plays a crucial role in inhibiting the neuroinflammatory response and mitochondrial reactive oxygen species production and has a neuroprotective effect against brain ischemia-reperfusion injury (I/R). In addition, *in vitro* experiments showed that miR-217 promotes the accumulation of histone deacetylase 5 (HDAC5) in the nucleus by targeting MEF2D, resulting in decreased expression of IL-10, thereby aggravating cognitive dysfunction after cerebral ischemia. Rao [23] found that the downregulation of miR-217 could alleviate oxygen-glucose deprivation reoxygenation (OGD/R)-induced neuronal damage by upregulating SIRT1 expression. Furthermore, a study found that elevated CB2 expression in circulating monocytes at 24 and 48 hours after cerebral ischemia was associated with elevated levels of miR-665, suggesting that miR-665 might regulate monocyte responses in ischemic stroke and play a role in sex [24]. Yang [25] found that miR-665 overexpression inhibited oxidative stress, promoted OGD/R-induced cerebral ischemia cell proliferation, and inhibited apoptosis, thus providing a potential therapeutic target for improving the outcomes in patients with cerebral ischemia.

KEGG enrichment analysis showed the enrichment of many cancer pathways, such as colorectal, endometrial, and prostate cancer pathways. Jiang et al. [26] found that approximately 15% of cancer patients were at risk of developing ischemic stroke (IS) later in life, and that 10% of IS hospitalized patients may have cancer simultaneously. In addition, these four cancer pathways were jointly enriched in four genes, CTNNB1, TCF7L2, NRAS, and KRAS, mainly involved in cell proliferation. CTNNB1 is a gene encoding the β-catenin protein [27]. Neutrophils transcriptionally upregulate CTNNB1 expression to compensate for Mdm2/p53-mediated β-catenin degradation induced by exposure to ischemic neurons [28]. Researchers used sequence quality array technology to genotype 533 patients with IS, 500 patients with coronary heart disease, and 531 healthy subjects, and found for the first time that the CTNNB1 polymorphism rs2953 was significantly associated with IS and coronary heart disease, and that its expression in IS patients is reduced, which is related to the important role of β-catenin protein in neuroprotection and stem cell differentiation [29]. TCF7L2 is located on chromosome 10q25.2, contains 17 exons and has a length of approximately 21.5 kb [30]. One study found that the SNP rs7903146 variant of TCF7L2 was associated with a significantly increased stroke prevalence in subjects with diabetes [31]. NRAS, the third member of the RAS family after relaying KRAS and HRAS in human neuroblastoma and fibrosarcoma cell lines, encodes a small GTPase that regulates cellularity by transducing membrane-localized receptor tyrosine kinase signals to the nucleus, regulating the cell cycle, proliferation, maturation, and differentiation [32]. KRAS is a member of the RAS subfamily [33]. KRAS is one of the TOP 10 hub genes of a stroke gene array, indicating that KRAS may be involved in the occurrence and development of stroke [34].

In the PPI network, the following eight core proteins were identified: SMAD4, HIF-1A, CDKN1B, CDK6, FOXO3, CTNNB1, KRAS, and NRAS. SMAD4 is a member of the SMAD family of encoded signal transduction proteins that are essential for maintaining tissue homeostasis and cell cycle regulation [35, 36]. Amini et al. [37] found that SMAD4 expression increased after IS, especially in those with the GG allele rs975903. H1F-1A is an HIF-α gene, which is expressed in all tissues [38]. HIF-1A is considered an important target for stroke treatment, and studies have demonstrated that inhibiting HIF-1A can increase the mortality and cerebral infarction size of stroke [39]. In addition, HIF1A is one of the core proteins in a network pharmacology study of IS [40]. Kovalevа [41] also found a variant of the HIF-1A gene C1772T polymorphism, which could be used as a molecular genetic molecule for predicting the risk of IS. CDK6 can activate the transcription of downstream genes involved in cell cycle regulation [42]. Zhao et al. [43] found that miR-424 inhibited the proliferation and activation of microglial cells by targeting cell cyclins, such as CDK6 and CDC25A, and exerted neuroprotective effects on the ischemic brain. In addition, a mouse experiment confirmed that miR-99a inhibited the expression of cyclin D1 and CDK6 following brain I/R injury, thereby regulating cell cycle progression and preventing apoptosis from reducing neuronal damage [44]. FOXO3 is a transcription factor that plays a central role in various cellular functions [45]. A study showed that FOXO3 is closely related to the human lifespan and that it may have a protective effect against neurodegeneration [46]. Furthermore, Del et al. [47] found that FOXO3 expression was increased in the cerebral penumbra of rats with cerebral ischemia, which could play a protective role against ischemic injury. In addition, FOXO3 overexpression induces autophagy in rat brain cells and reduces infarct size after stroke [48]. CDKN1B, also known as p27Kip1, acts as a regulator of the cell cycle and negatively regulates cell proliferation [49].

CTNNB1, KRAS, and NRAS are also three intersecting genes in the cancer pathway in this study, indicating that they may be key target genes regulating the development of AIS.

This study has some limitations. Since this is a hospital-based case-controlled study, not population-based, it might have been affected by selection bias. Furthermore, the specific mechanism of hsa_circ_0004099 regulating AIS is not clear, and animal and cell experiments were not conducted to explore this specific mechanism. Therefore, the mechanism by which hsa_circ_0004099 regulates AIS remains to be determined.

In conclusion, this study demonstrated that patients with AIS have low expression of hsa_circ_0004099. The NIHSS score and infarct time were negatively correlated with the expression levels of hsa_circ_0004099, suggesting that hsa_circ_0004099 may be a potential therapeutic target for improving AIS outcomes. Further research showed that the AUC of hsa_circ_0004099 is 92.3%, indicating a good predictive value for AIS. Hence, hsa_circ_0004099 is a potential tool for the diagnosis and prognosis of AIS with significant clinical implication.

## Supporting information

S1 Fig Raw images of Figs 1–8. (PDF)

S1 Table Differentially expressed circRNAs. (XLSX)

S2 Table Intersection of miRNAs. (XLSX)

S3 Table Intersection of mRNAs. (XLSX)

S4 Table circRNA-miRNA-mRNA networks date. (XLSX)

S5 Table GO and KEGG enrichment analysis data. (XLSX)
