# Correction: Bioinformatic analysis and clinical diagnostic value of hsa_circ_0004099 in acute ischemic stroke

**DOI:** 10.1371/journal.pone.0292779

**Published:** 2023-10-05

**Authors:** Jiqing Zheng, Shuiming Luo, Yaobin Long

There are errors in the Funding section. The correct Funding statement is: Dr Yaobin Long was supported by the following three funding sources: Guangxi Medical and health key discipline construction project (Guiwei Kejiao Fa [2022] No. 4), Natural Science Foundation of Guangxi Province (2022GXNSFAA035511), and Guangxi Science and Technology Major Project (Gui Ke AA17204017-5).
