# Supplementary material for: Bioinformatic analysis and clinical diagnostic value of hsa_circ_0004099 in acute ischemic stroke
Source: PLoS One. 2022 Nov 18;17(11):e0277832. doi: 10.1371/journal.pone.0277832 (PMC9674149; doi:10.1371/journal.pone.0277832)
Supplement: S1 Fig — (PDF) [file pone.0277832.s001.pdf]

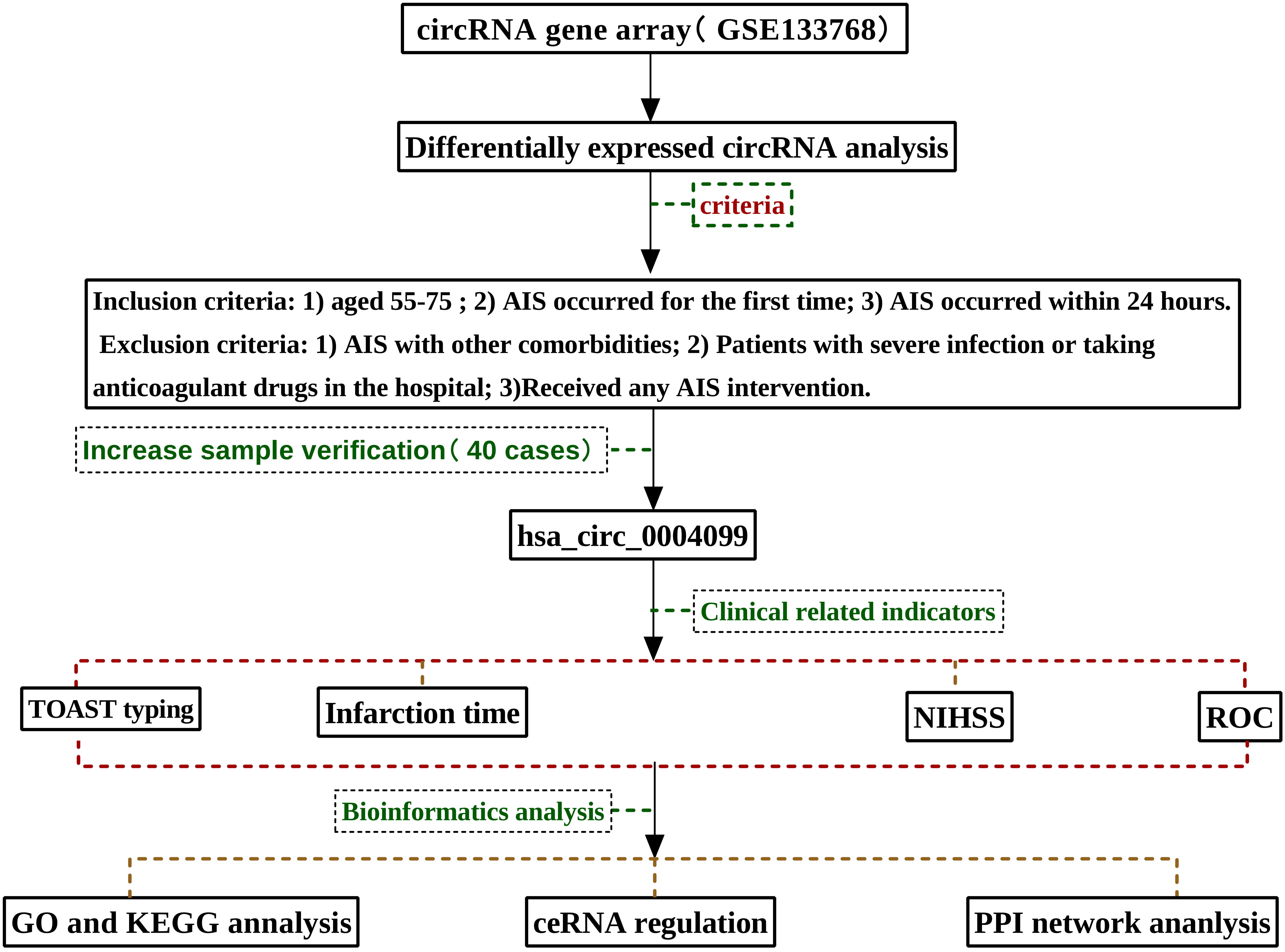
circRNA gene array（GSE133768）
Differentially expressed circRNA analysis
criteria
Inclusion criteria: 1) aged 55-75 ; 2) AIS occurred for the first time; 3) AIS occurred within 24 hours.
Exclusion criteria: 1) AIS with other comorbidities; 2) Patients with severe infection or taking anticoagulant drugs in the hospital; 3)Received any AIS intervention.
Increase sample verification（40 cases）
hsa_circ_0004099
Clinical related indicators
TOAST typing
Infarction time
NIHSS
ROC
Bioinformatics analysis
GO and KEGG annalysis
ceRNA regulation
PPI network ananlysis

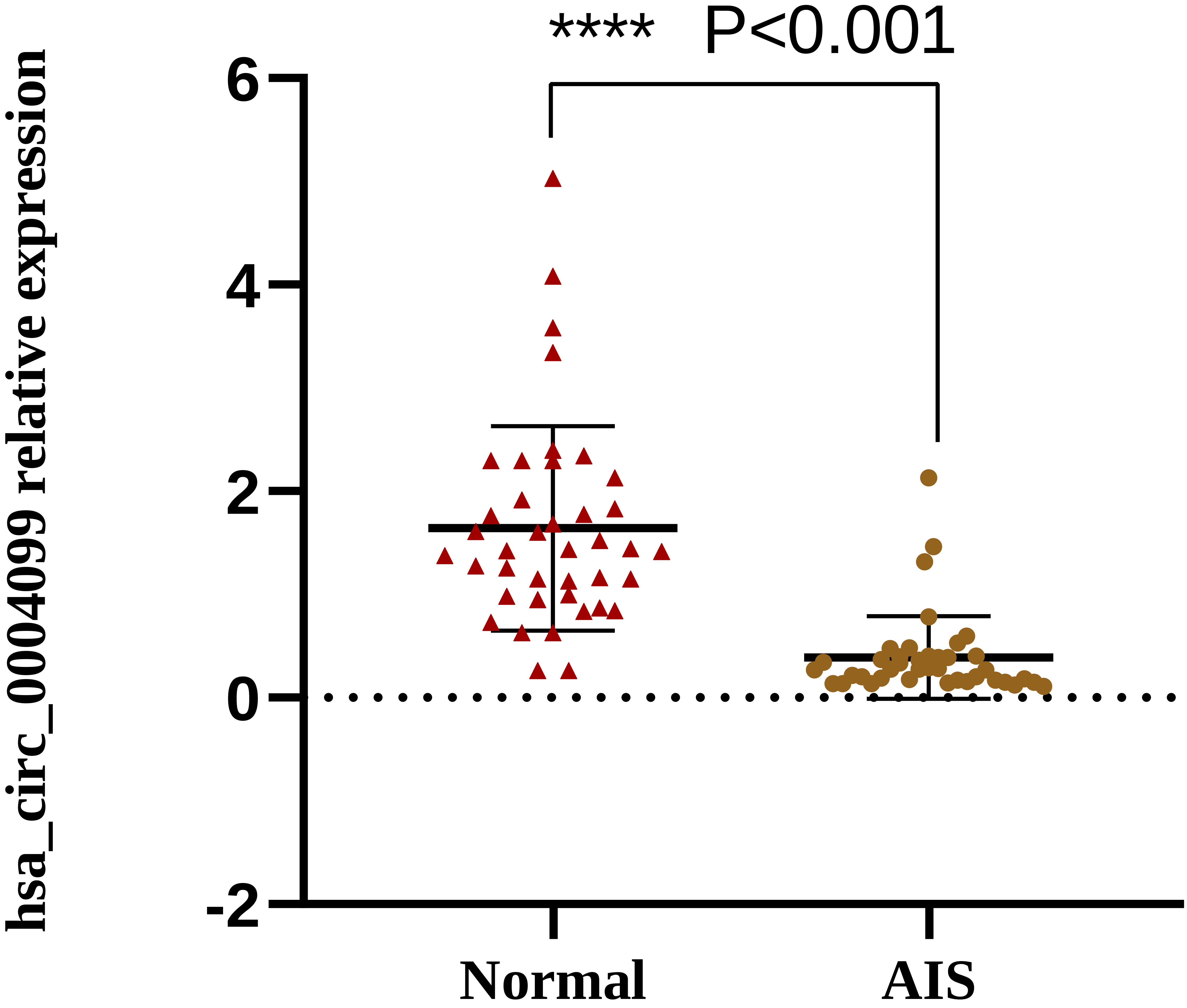

**** P<0.001
hsa_circ_0004099 relative expression
6
4
2
0
-2
Normal
AIS

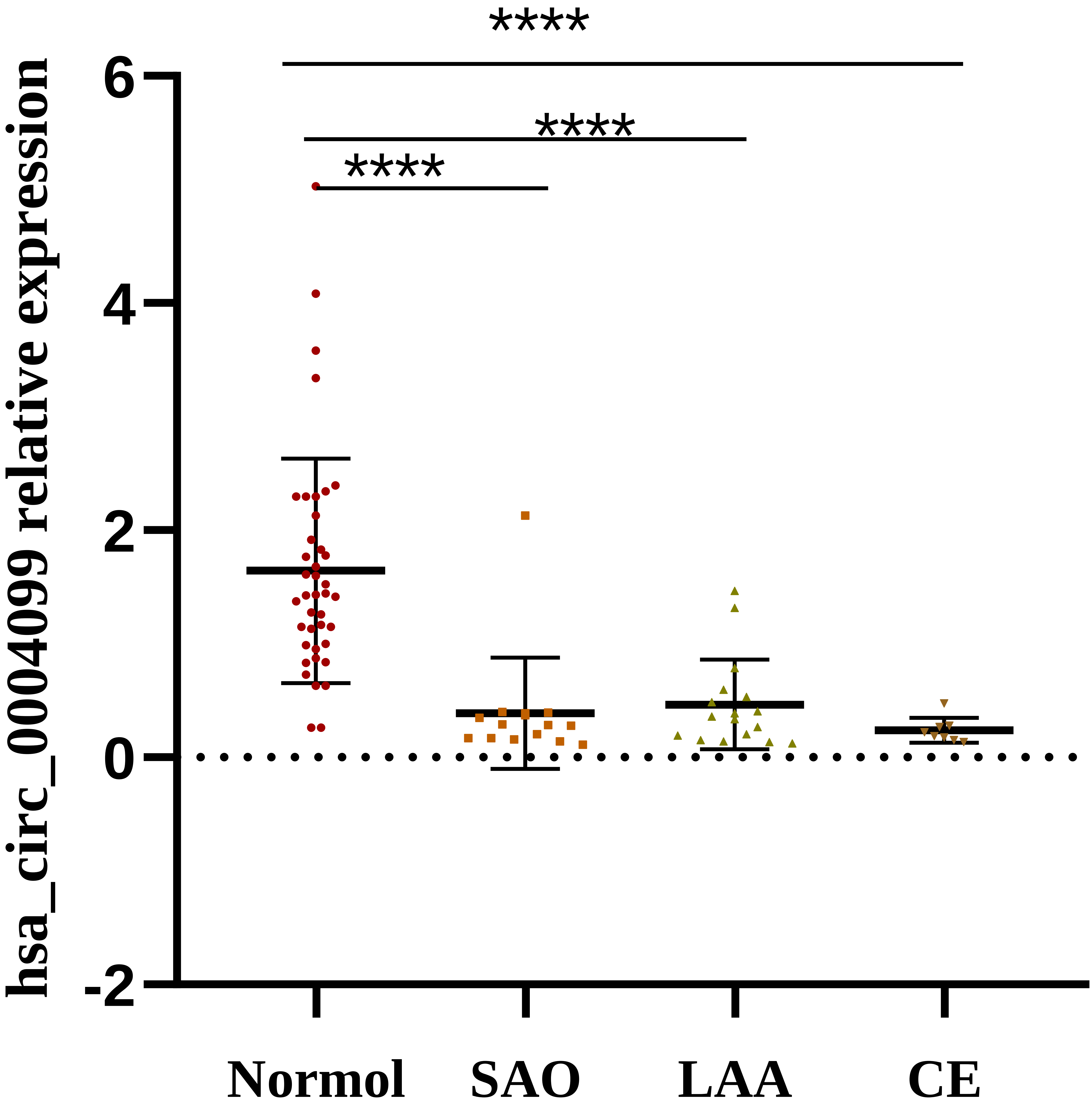

****
****
****
hsa_circ_0004099 relative expression
6
4
2
0
-2
Normol
SAO
LAA
CE

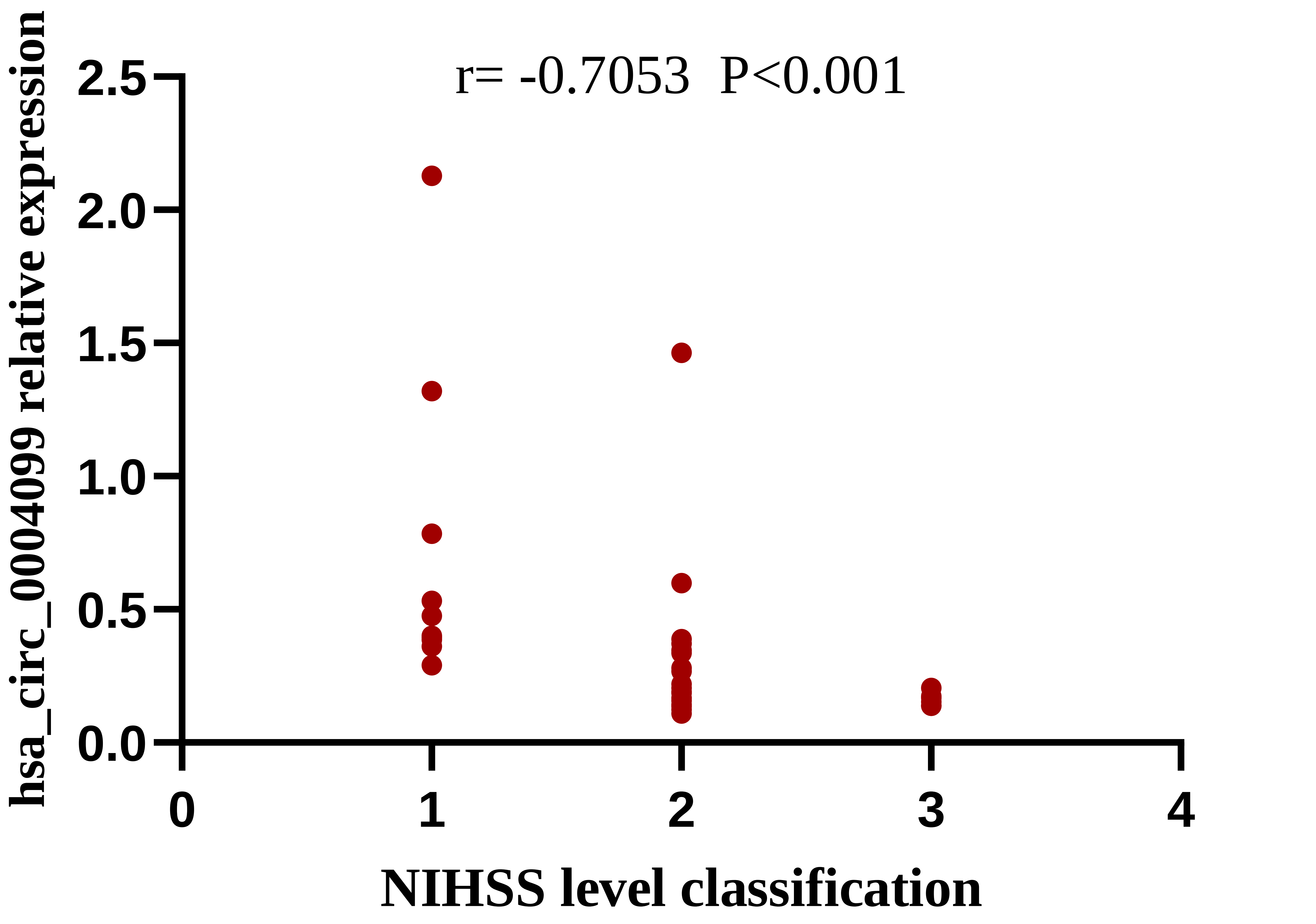

r= -0.7053 P<0.001
hsa_circ_0004099 relative expression
2.5
2.0
1.5
1.0
0.5
0.0
0
1
2
3
4
NIHSS level classification

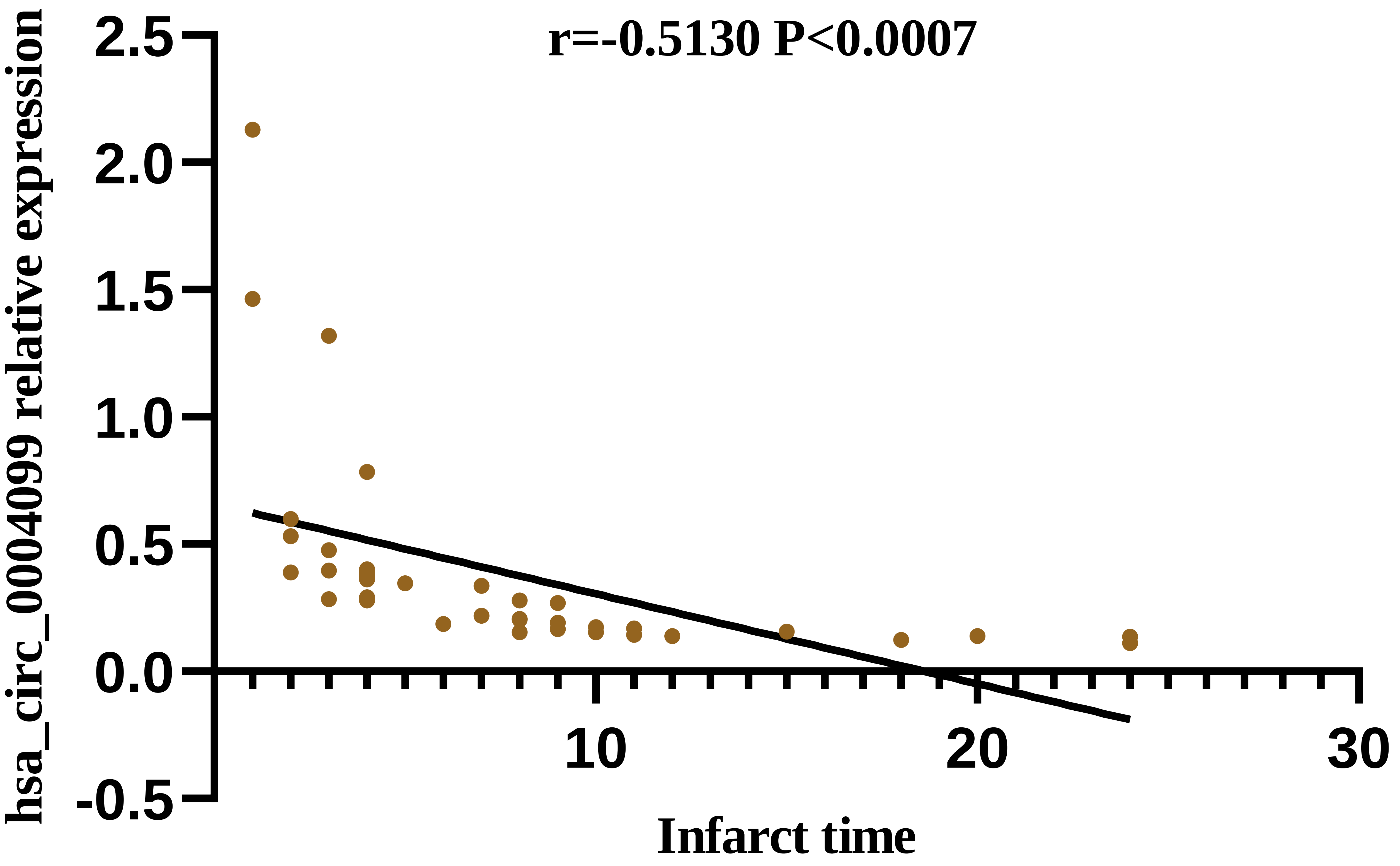
r=-0.5130 P<0.0007
hsa_circ_0004099 relative expression
2.5
2.0
1.5
1.0
0.5
0.0
-0.5
10
20
30
Infarct time

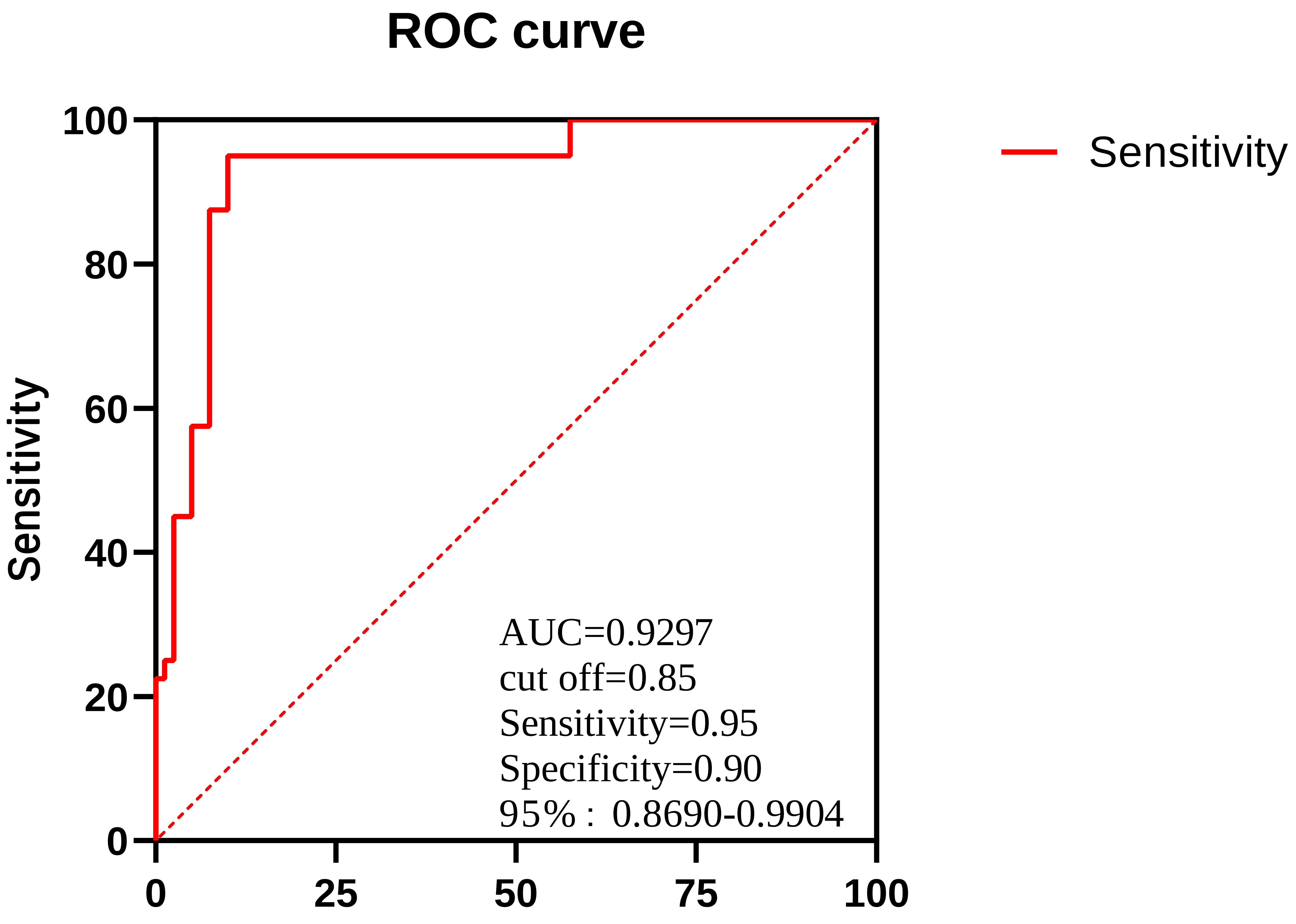
ROC curve
Sensitivity
100
80
60
40
20
0
0
25
50
75
100
AUC=0.9297
cut off=0.85
Sensitivity=0.95
Specificity=0.90
95%：0.8690-0.9904
Sensitivity

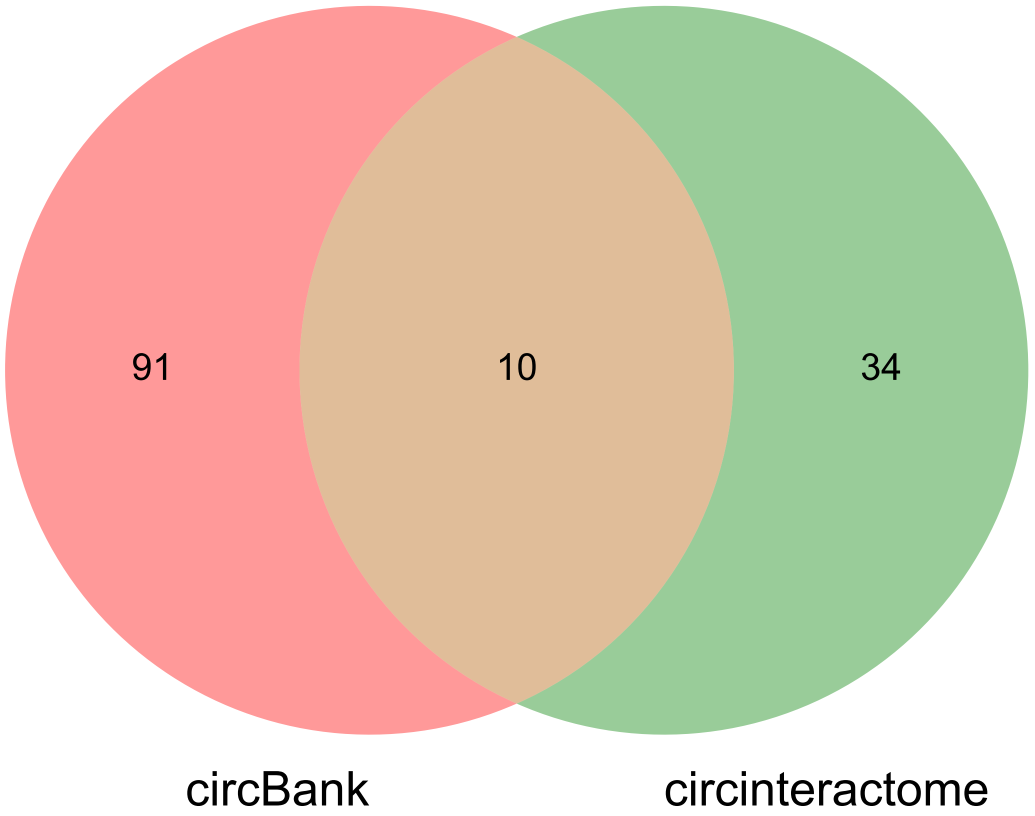
91
10
34
circBank
circinteractome

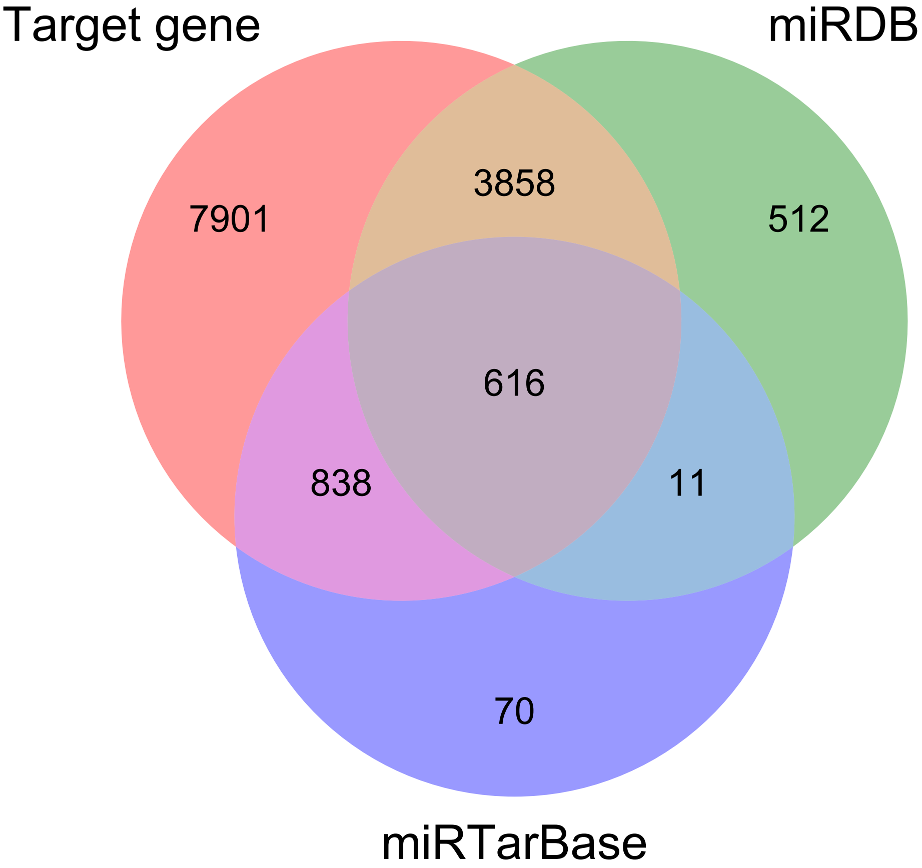
Target gene
miRDB
7901
3858
512
616
838
11
70
miRTarBase

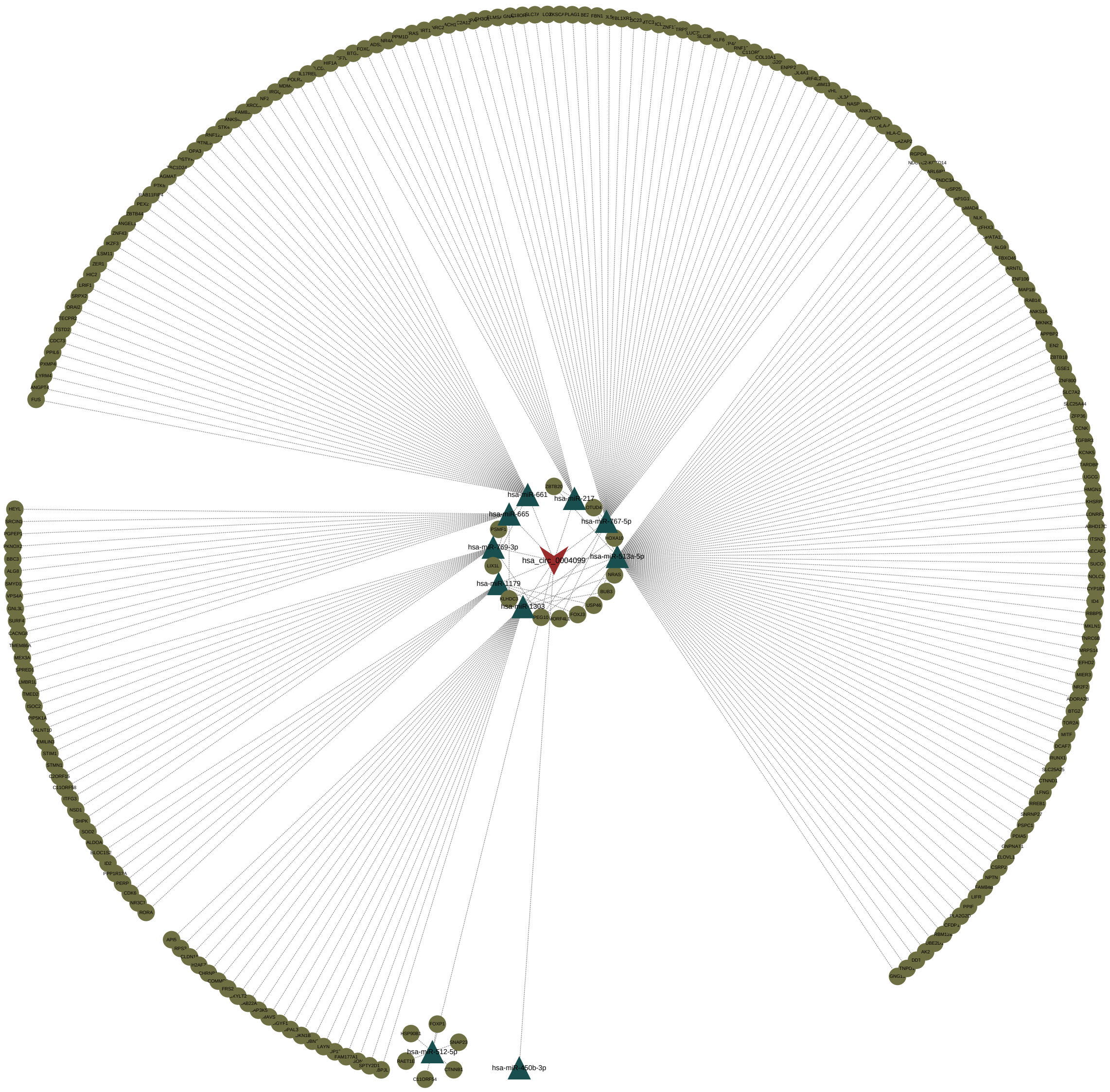
hsa_circ_0004099
hsa-miR-661
hsa-miR-665
hsa-miR-767-5p
hsa-miR-1179
hsa-miR-512-5p

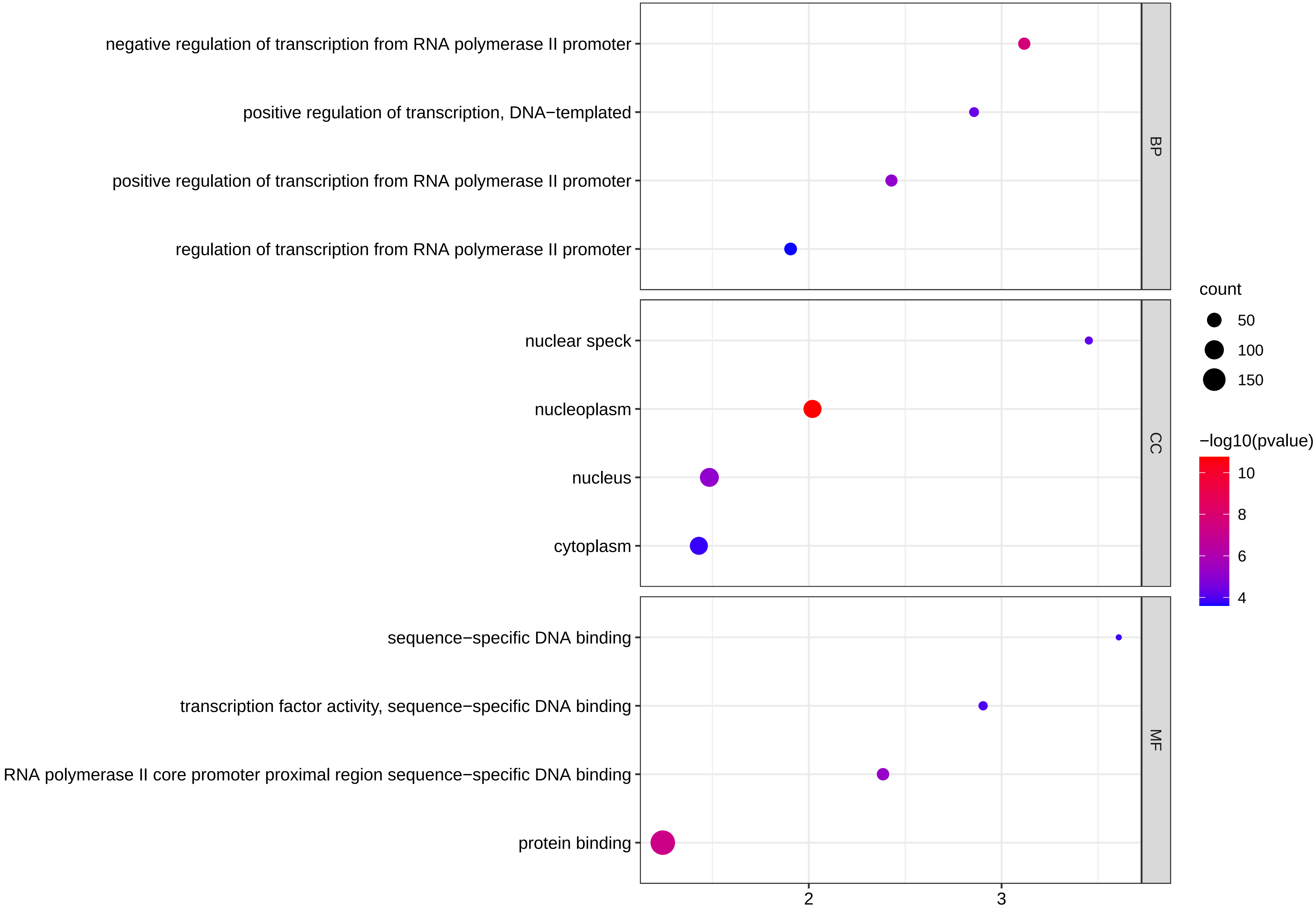

negative regulation of transcription from RNA polymerase II promoter
positive regulation of transcription, DNA−templated
positive regulation of transcription from RNA polymerase II promoter
regulation of transcription from RNA polymerase II promoter
BP
nuclear speck
nucleoplasm
nucleus
cytoplasm
CC
sequence−specific DNA binding
transcription factor activity, sequence−specific DNA binding
RNA polymerase II core promoter proximal region sequence−specific DNA binding
protein binding
MF
2
3
count
50
100
150
−log10(pvalue)
10
8
6
4

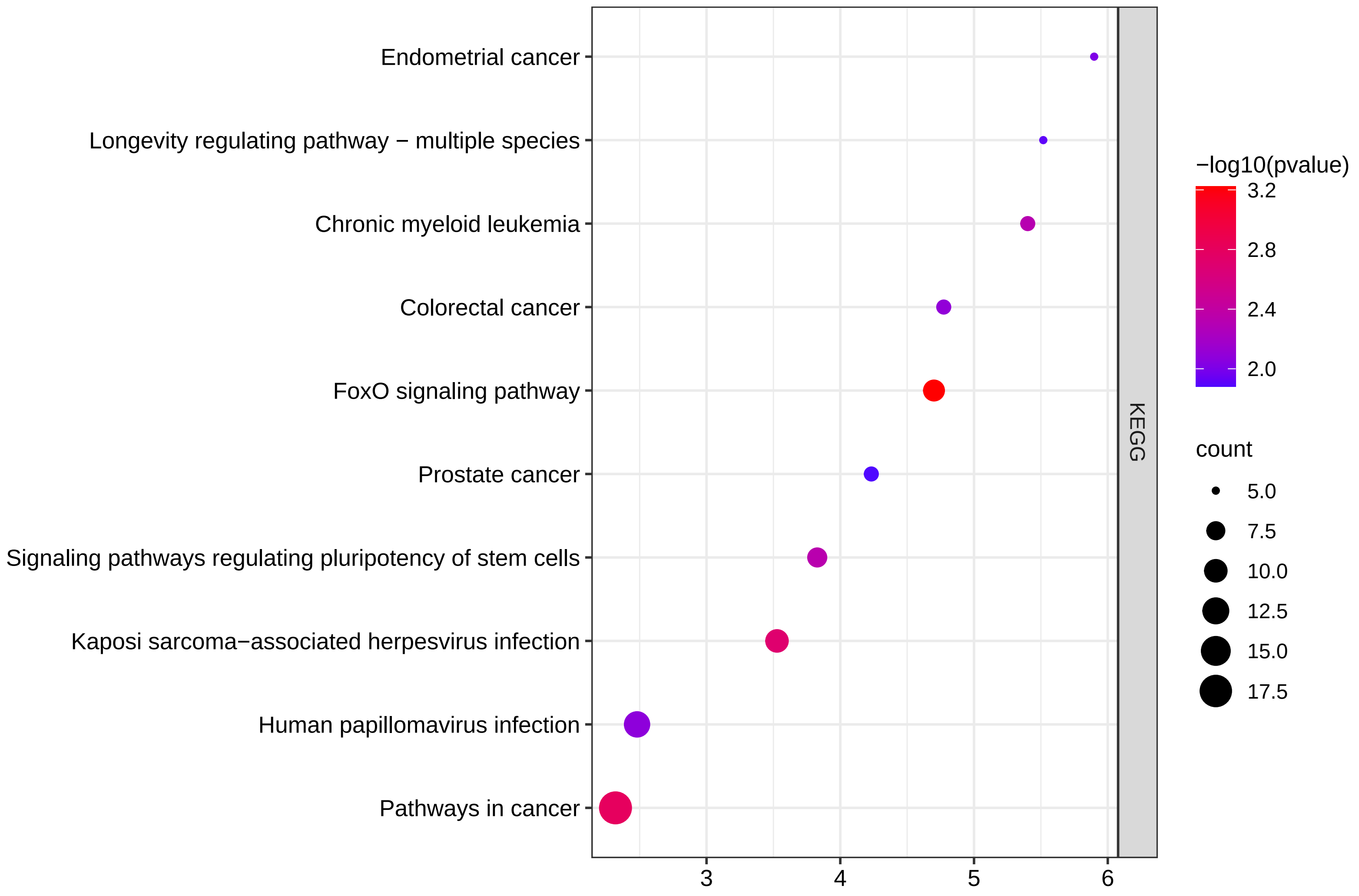
Endometrial cancer
Longevity regulating pathway − multiple species
Chronic myeloid leukemia
Colorectal cancer
FoxO signaling pathway
Prostate cancer
Signaling pathways regulating pluripotency of stem cells
Kaposi sarcoma−associated herpesvirus infection
Human papillomavirus infection
Pathways in cancer
3
4
5
6
KEGG
−log10(pvalue)
3.2
2.8
2.4
2.0
count
5.0
7.5
10.0
12.5
15.0
17.5

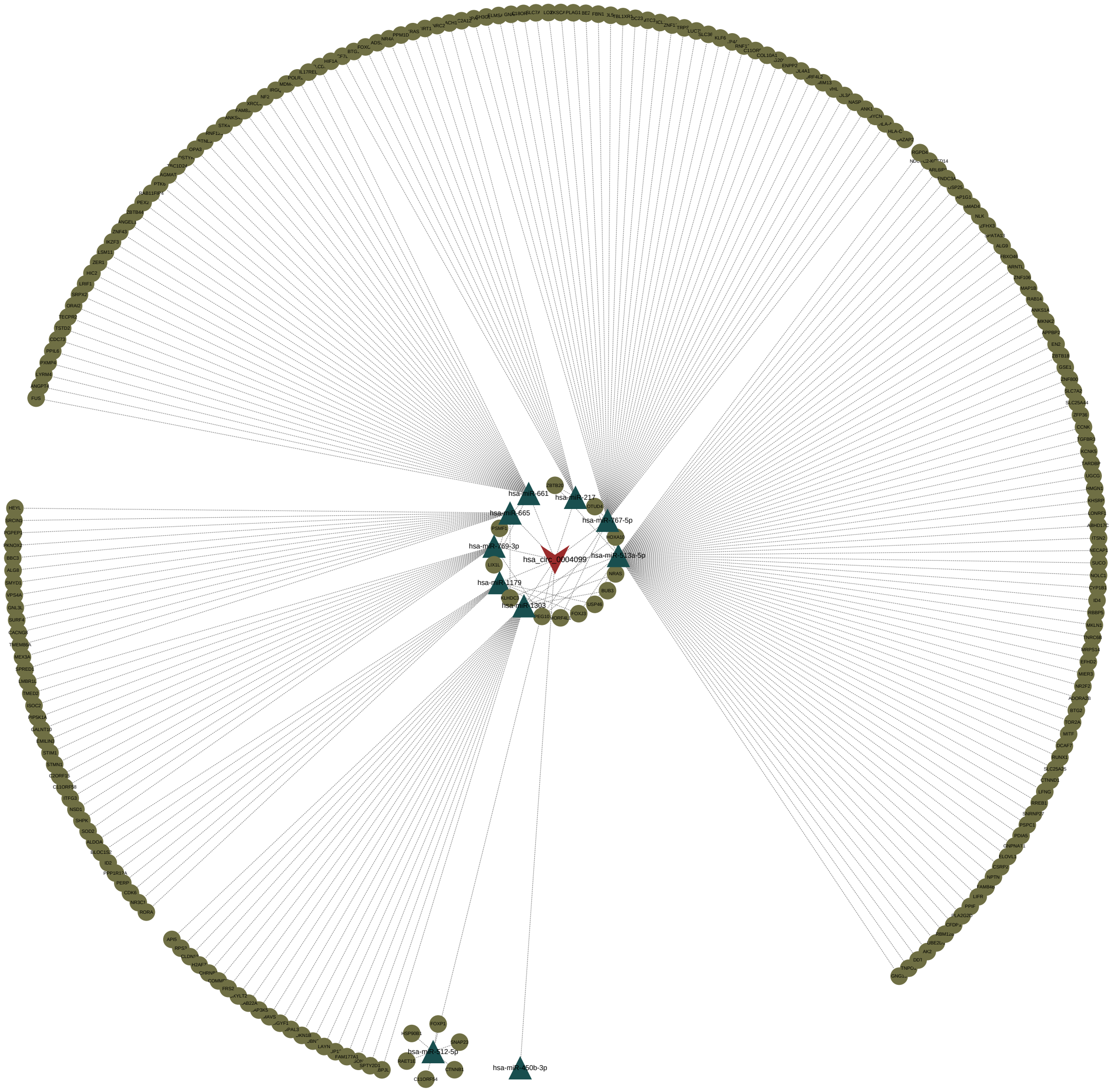
hsa_circ_0004099
hsa-miR-661
hsa-miR-217
hsa-miR-665
hsa-miR-767-5p
hsa-miR-769-3p
hsa-miR-513a-5p
hsa-miR-1179
hsa-miR-1303
hsa-miR-512-5p
hsa-miR-450b-3p
ZBTB20
OTUD4
PSMF1
HOXA10
LIX1L
NRAS
BUB3
KLHDC3
USP46
FOXJ3
PEG10
MORF4L1
FOXP1
HSP90B1
SNAP23
RAET1E
CTNNB1
C11ORF54
SPTY2D1
